# Pan-Cancer Analysis of TLE3 Revealed Its Value in Tumor Microenvironment and Prognosis

**DOI:** 10.1155/2022/4085770

**Published:** 2022-11-21

**Authors:** Tianyu Li, Ranran Liu, Guohong Zhang, Yejuan Jia, Lijia Pan, Yunfeng Li, Chunsheng Jia

**Affiliations:** ^1^School of Acupuncture, Moxibustion and Tuina, Hebei University of Chinese Medicine, Shijiazhuang 050200, China; ^2^Department of Biochemistry and M Biology, College of Basic Medicine, Hebei University of Chinese Medicine, Shijiazhuang 050200, China

## Abstract

**Background:**

Transducin-like enhancer of split 3 (TLE3), a member of the TLE gene family, is related to tumor genesis and progression. However, whether TLE3 played a crucial role in the whole pan-cancer remained unknown.

**Methods:**

Comprehensive analysis of TCGA, GEO, and GTEx data with an online tool, and *R* language was performed to explore the relationship of TLE3 expression between prognosis, gene mutation, protein phosphorylation, DNA methylation, tumor microenvironment, and related pathways in 33 tumors.

**Results:**

TLE3 was high-expressed in most tumors, and TLE3 expression and the prognosis of some tumor types were significantly correlated. The level of TLE3 expression in 33 cancer types was closely associated with DNA methylation. High-level phosphorylation sites of Tle3, such as S267 and S217, may promote cancers. In terms of the tumor microenvironment, TLE3 affected a wide variety of cancers, especially PRAD and LIHC, and TLE3 may act on them via immune-related pathways.

**Conclusions:**

The current work provided the first comprehensive investigation of TLE3 in a pan-cancer study, highlighting the role of TLE3 in the tumor immune microenvironment, and also determined the potential of TLE3 as a prognostic, immunotherapy response, and diagnostic biomarker in many cancers. However, the present results were preliminary and required further validation as this study was based on bioinformatics analyses.

## 1. Introduction

Cancer is one of the most challenging diseases in clinical treatment, and its threat to human health is becoming increasingly serious [1]. Many oncogenic and tumor suppressor genes are implicated in cancer initiation and progression. Constantly developing techniques and bioinformatics databases would allow us to analyze more genes of interest in pan-cancer.

As a conserved family of corepressor proteins, the transducin-like enhancer of split (TLE) is present in multiple animals, including mice and humans. The TLE protein family plays a key role in the entire life cycle of animals, including lateral inhibition, segmentation, sex determination, and eye and pancreatic development, and regulates basic processes such as metabolism through interacting with multiple pathways [2].

TLE3 as one of the TLE family proteins is a transcriptional inhibitory homolog of the Groucho protein [3], which is a part of the Drosophila Notch signaling cascade and may be expressed periodically during the *M* phase of the cell cycle [4]. It cooperates with the transcription factor HHEX to promote memory B cell development [5]. TLE3 has been shown to have inhibitory or promoting effects on different tumors [6, 7]and is a predictive marker for response to taxane-containing regiments in breast and nonserious ovarian cancer [8, 9]. However, TLE3 has previously been studied in only a few cancer types, its role in other types of cancer remains unclear, and the pathogenesis or impact on the survival of different cancers has not been elucidated.

## 2. Materials and Methods

### 2.1. Gene Ontology Analysis

Genomic localization information for the TLE3 gene was obtained from the UCSC genome browser on humans (GRCh37/hg19) (https://genome.ucsc.edu/) [10]. The analysis of TLE3 protein conserved functional domain in multiple species was analyzed by clicking the “HomoloGene” button of the NCBI(https://www.ncbi.nlm.nih.gov/) and then the “constraint-based multiple sequence alignment online tool.” The phylogenetic tree of TLE3 in various species was mapped by the “constraint-based multiple sequence alignment online tool.” In addition, we accessed the data of the TLE3 mRNA expression in various cells and tissues under physiological conditions using an online HPA database (https://www.proteinatlas.org/humanproteome/pathology).

### 2.2. mRNA and Protein Expression Analysis

The differentially expressed TLE3 between various tumors and corresponding normal tissues were determined by clicking on the “Gene_de” button on TIMER2 web (https://timer.cistrome.org/). For some cancers without normal tissues information (e.g., LGG), the GEPIA2 web server (https://gepia2.cancer-pku.cn/#analysis) [11] was applied, and we clicked the “Match TCGA normal and GTEx data” to acquire a chart of TLE3 expression contrast between these tumor tissues and the corresponding normal tissues using the GTEx database. The TLE3 protein expression was obtained from the UALCAN portal (https://ualcan.path.uab.edu/analysis-prot.html) using the “CPTAC” dataset [12]. Then, we determined the levels of TLE3 protein expression between primary tumors and corresponding normal tissues. With the HPA database (HumanProteinAtlas), we acquired IHC (immunohistochemical) images to show the protein expression of TLE3 in tumors and corresponding normal tissues. Next, by the “Pathological Staging” module in GEPIA2, the different pathological (stages I, II, and III) stages of the tumor were obtained.

### 2.3. Survival Analysis

OS (overall survival) and DFS (disease-free survival) data of the TLE3 gene were acquired in the “Survival Map” option of GEPIA2 [1], and the survival graphs were then produced in the “Survival Analysis” module. We then performed analysis and visualization using the uniformly standardized pan-cancer dataset according to the TCGA database in the *R* environment of survival and survminer package. We used multivariate Cox regression analysis to study the relationship between TLE3 expression and OS, DSS (disease-specific survival), and PFI (progression-free interval). Results may vary due to different algorithms. To further validate the association between TLE3 expression and cancer survival, the Kaplan–Meier plotter (https://kmplot.com/analysis/) was used to analyze the OS, DMFS (distant metastasis-free survival), RFS (relapse-free survival), PPS (postprogression survival), FP (first progression), DSS, and PFS (progression-free survival).

### 2.4. Genetic Alteration Analysis

We obtained the mutations in the TLE3 gene using the cBioPortal web(https://www.cbioportal.org/) [13, 14], including the frequency of mutations, mutation types, CNA (copy number alteration), mutation site information, and 3D protein structure maps of the TCGA tumors. Then, patients with and without TLE3 gene mutations were compared for differences in terms of overall, disease-free, progression-free, and disease-free survivals and Kaplan–Meier plots. The results were shown by log-rank *P*-value.

### 2.5. DNA Methylation Analysis

Based on the TCGA database, the UALCAN online tool was used to evaluate TLE3 gene promoter methylation levels between the primary tumors and corresponding normal tissues [15].TLE3 DNA methylation levels were determined through the MEXPRESS website. Based on the TGGA data, we used the *R* package to plot the association between TLE3 levels and DNA methyltransferases.

### 2.6. Protein Phosphorylation Analysis and Immunotherapeutic Response of TLE3

Expression information of TLE3 protein phosphorylation was obtained from the UALCAN portal. We obtained the relationships between TLE3 mRNA expression and TMB, MSI analysis data from SangerBox (https://sangerbox.com/Tool). We integrated the data and log-transformed each expression value, which was then modified using Microsoft PowerPoint software to generate images. We also used ggplot2 [version 3.3.3] to visualize the mismatch repair gene proteins (MLH1, MSH2, MSH6, PMS2, and EPCAM) based on the TGCA database.

### 2.7. Immunocorrelation Analysis of TLE3

Immunocorrelation analysis of TLE3 was downloaded from TISIDB (https://cis.hku.hk/TISIDB/), a storage website integrating rich human cancer datasets in the TCGA database [16]. In addition, we also obtained the immune cell infiltration of TLE3 in the MCPcounte algorithm from SangerBox.

### 2.8. TLE3-Related Gene Enrichment Analysis

The experimentally identified TLE3 and binding proteins were available from the STRING website [17] (https://string-db.org/), and a protein-protein interaction (PPI) network map was generated. We exported short tabular text in TSV format and substituted it into Cytoscape 3.9.1 software to obtain the visual network diagram. The first 100 target genes associated with tle3 were obtained by selecting “TCGA + GTEx” in the “similar gene detection” function of GEPIA2. A scatter plot of the association between TLE3 and the first 5 related genes was then generated, and a heat map of the correlation between these 5 genes and each tumor in different algorithms was obtained on the TIMER2 website. With jvenn (interactive Venn diagram viewer) [18], we compared TLE3 binding genes and interacting genes by cross-tabulation analysis to obtain the intersection. Analysis of functional enrichment for genes interacting with TLE3, including BP (biological process), CC (cellular component), and MF (molecular function), were performed using the “clusterProfiler” *R* package, and KEGG pathway (https://www.kegg.jp/kegg/kegg1.html) was obtained from the DAVID database (https://david.ncifcrf.gov) [19]. After downloading the enrichment pathway analysis data from the DAVID database, the Cytoscape 3.9.1 software was inserted to obtain a visual network map using the enrichment map plugin. GSEA analysis of TLE3 data on BRCA and LIHC was obtained from LinkedOmics (http://linkedomics.org/) [20].

### 2.9. Statistic Analysis

We used Pearson's or Spearman's coefficient analysis to investigate the correlations between the variables. Continuous variables fitting a normal distribution between binary groups were compared using a *t*-test. Otherwise, the Mann–Whitney *U* test was used. Categorical variables were compared by the chi-squared test or Fisher's exact test. All the statistical data analyses were implemented using *R* software, version 3.6.3. Two-tailed *P* < 0.05 was considered statistically significant.

## 3. Results

### 3.1. Gene Ontology Analysis

**Figure 1:**
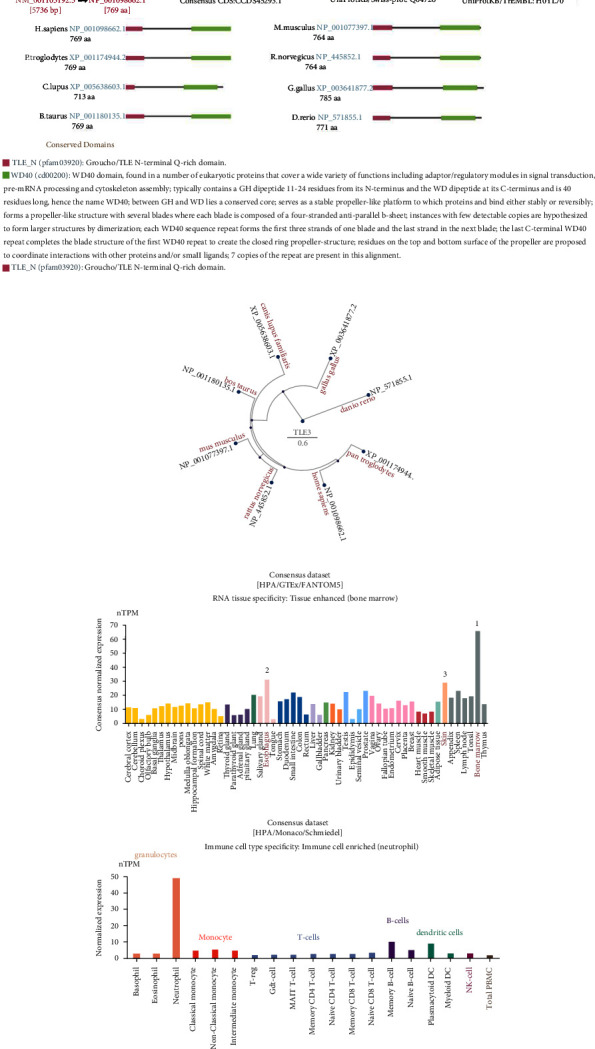
The structural characteristics of TLE3 in different species and its expression in different cells, tissues under normal physiological states. (a) Genomic positioning of the human Tle3 gene and conserved domains of the TLE3 protein among different species, (b) phylogenetic tree of TLE3 in different species, and (c) expression levels of TLE3 in different tissues and cells.

Firstly, we discussed the basic information of TLE3 (NM_001105192.3 for mRNA or NP_001098662.1 for protein, Figure 1(a)) in humans. TLE3 is located at locus 70047790–70098176 on chromosome 15. Among animals including *H. sapiens*, *P. troglodytes,* and *M. mulatta*, the TLE3 protein structure is conserved and commonly made up of the TLE_N (pfam03920) and WD40 (cd00200) domains. The data of the phylogenetic tree (Figure 1(b)) shows the evolution relationship relation of the TLE3 protein in multiple species.

We then explored the TLE3 expression in normal human tissues. According to the HPA, GTEx, and FANTOM5 consensus dataset, the top three TLE3 expressions were bone marrow, esophagus, and skin (Figure 1(c)). TLE3 is expressed in all tissues, regions, and cell types, showing an enhanced RNA tissue specificity. Analysis of TLE3 expression in various immune cells from the combination of HPA, Monaco, and Schmiedel datasets also showed enriched RNA blood cell type specificity (Figure 1(d)). However, there was no information on blood protein concentration in plasma about TLE3.

### 3.2. mRNA and Protein Expression Analysis

**Figure 2:**
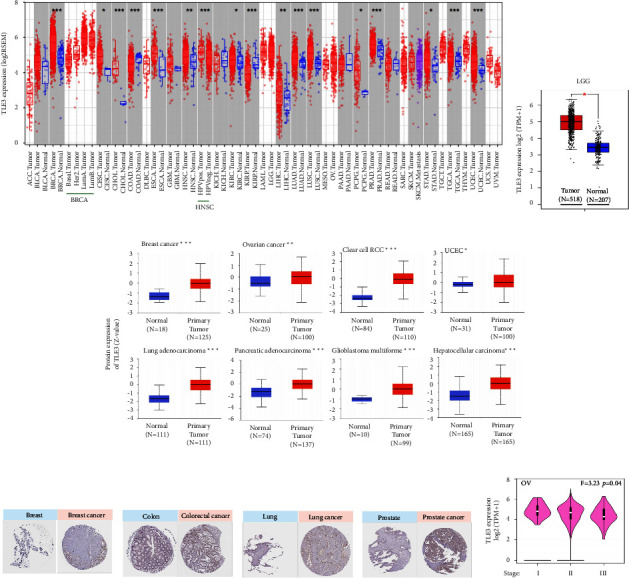
mRNA expression states and protein level of TLE3 in human tumors. (a) mRNA expression level of TLE3 in tumor and normal tissues as visualized by TIMER2.  ^*∗*^*P* < 0.05;  ^*∗∗*^*P* < 0.01;  ^*∗∗∗*^*P* < 0.001, (b) TLE3 mRNA expression level comparison in LGG (TCGA project) relative to the corresponding normal tissues (GTEx database).  ^*∗*^*P* < 0.05, (c) the total protein level of TLE3 in normal and tumor tissue visualized by CPTAC.  ^*∗∗∗*^*P* < 0.001, (d) comparison of TLE3 gene expression between normal and tumor tissues shown by immunohistochemistry images, and (e) the stage-dependent expression level of TLE3. The main pathological stages (stage I, stage II, and stage III) of OV were assessed and compared by TCGA data.

We applied TIMER2 to explore the TLE3 mRNA expression across 33 cancers. As Figure 2(a) shows, the level of TLE3 mRNA expression in the 16 types of tumor tissues was higher than the corresponding control tissues. Normal tissue data from the GTEx dataset were used as a control, we further evaluated the difference in TLE3 mRNA expression between LGG control tissues and tumor tissues (Figure 2(b), *P* < 0.05). However, no significant differences were shown in other cancers (Figure S1(a)). Proteins are the macromolecules that ultimately perform biological functions in the human body, and have expression closer to phenotypes, allowing for more real-time monitoring of disease progression. Therefore, based on the CPTAC dataset, we determined the TLE3 protein expression levels in 33 cancers. The results showed that total protein expression of TLE3 was elevated in the primary tissues of breast cancer, clear cell RCC, lung adenocarcinoma, pancreatic adenocarcinoma, glioblastoma multiforme, hepatocellular carcinoma, ovarian cancer, and UCEC than in normal tissues. As shown in Figure 1(c), RNA levels (transcription levels) and protein levels (translation levels) of TLE3 were consistent in part, and the results of the two databases validated each other. In addition, to further verify the protein level of TLE3 expression, we compared the IHC results from the HPA database, which was also in line with gene expression data from the TCGA (Figure 2(d)). Normal breast, colon, lung, and prostate TLE3 IHC staining of tissues were negative or moderate, while that of tumor tissues were moderate or strong (Figure 2(e)). We also analyzed the relation between the mRNA expression of TLE3 and the pathological stages of cancer, OV showed significant differences (Figure 2(e)*P* < 0.05), while others did not (Figures S1(b)–S1(g)).

### 3.3. Survival Analysis

**Figure 3:**
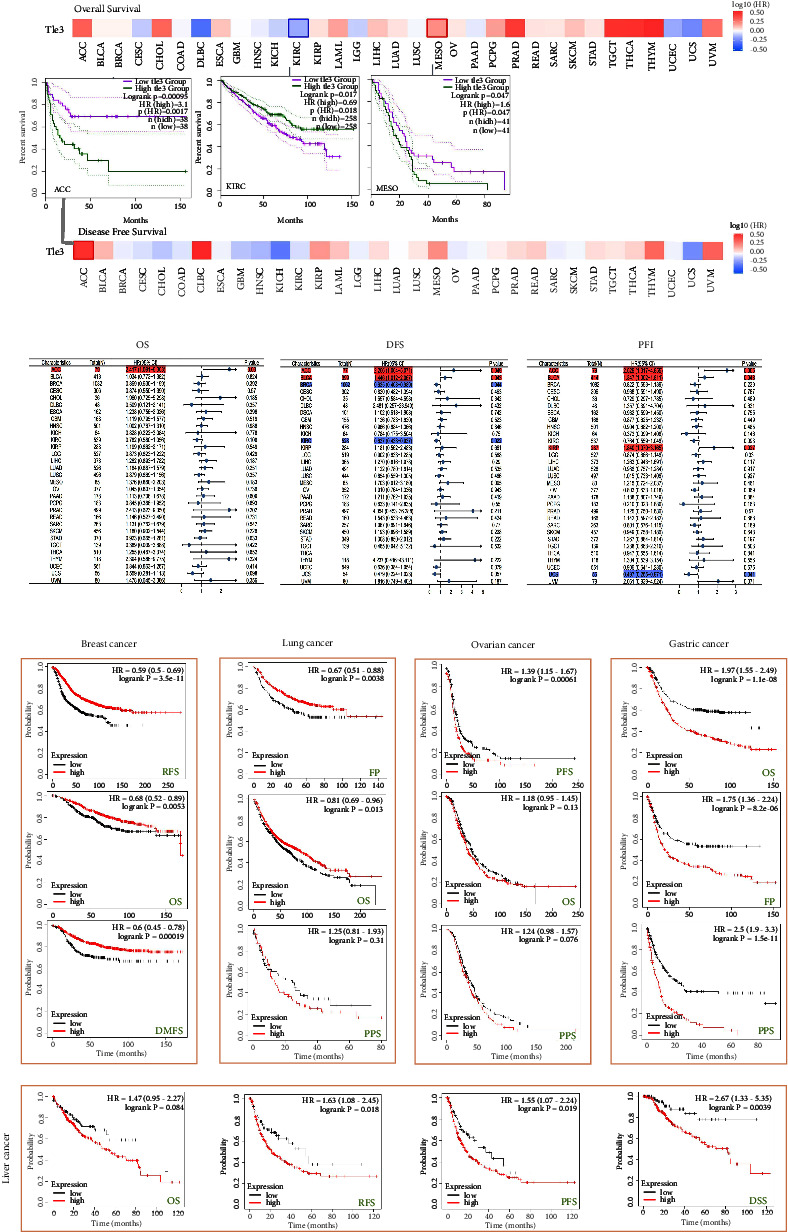
Association between Tle3 mRNA expression and survival of cancer in TCGA. (a) Overall survival and disease-free survival were shown by the GEPIA2 tool, (b) the multivariate Cox regression analysis of TLE3 mRNA expression levels in cancers was performed, and (c) the relationship between the TLE3mRNA expression level and the tumor patients' survival, is shown by the Kaplan Meier plotter.

As TLE3 was overexpressed in pan-cancer, we further explored its prognostic profile. Low expression of *TLE3* was connected to the poor OS of KIRC, whereas high *TLE3* expression was associated with poor OS of MESO and poor DFS of ACC, according to the GEPIA2 database (Figure 3(a)). We then studied the relationship between TLE3 mRNA expression and survival of ACC, BLCA, BRCA, KIRC, KIRP, and UCS through multivariate Cox regression analysis (Figure 3(b)). Moreover, survival data were analyzed using the Kaplan–Meier mapping tool, and a correlation was found between low TLE3 expression and poor prognosis of breast cancer in RFS, OS, and DMFS (Figure 3(c)). Additionally, low expression of TLE3 was associated with poor prognosis of FP and OS in lung cancer. By contrast, the high-expressed mRNA level of TLE3 was connected with poor PFS (progress-free survival) of ovarian cancer, poor OS, FP, and PPS of gastric cancer, and poor RFS, PFS, and DSS of liver cancer. These data suggested that the expression of TLE3 had different prognostic effects in different tumors.

### 3.4. Genetic Alteration Analysis

**Figure 4:**
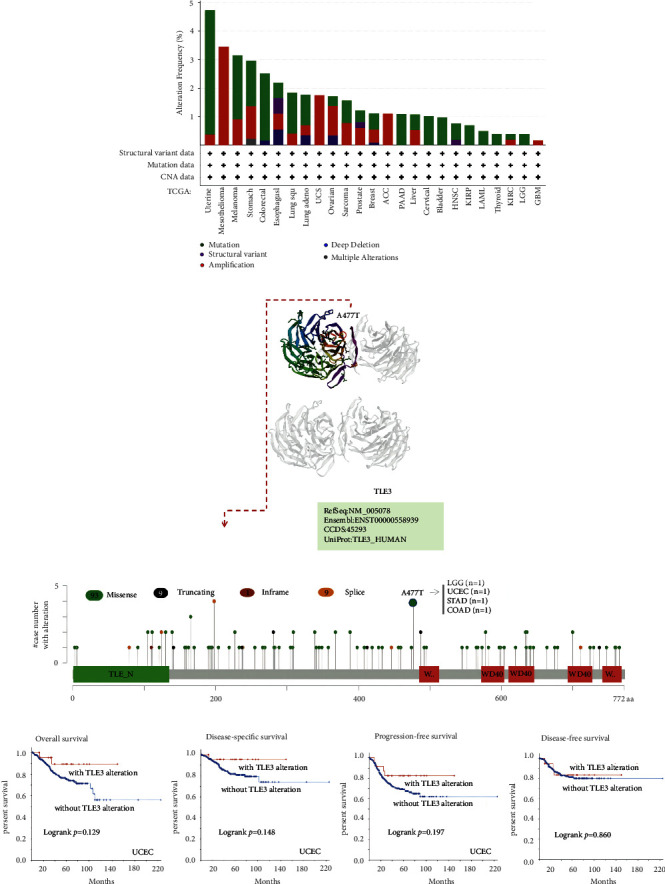
Genetic alteration analysis of TLE3 using the cBioPortal tool.

Genetic mutations have an important influence on cancer development, and these mutated genes may also be used as effective therapeutic targets. Therefore, we also observed the state of *TLE3* genetic alteration in different tumor samples. The frequency of alteration in TLE3 was the highest (>4%) in patients with a “mutation” primary type of uterine tumor. It should be noted that all cases of genetically altered mesothelioma (more than 3% frequency) had amplification of *TLE3* (Figure 4(a)). More information is shown in Figure 3(b), including the types, sites, and caseload of the mutant TLE3 gene. The data demonstrated that the main type of TLE3 genetic alteration was missense mutation, and A477T alteration was detected in LGG, UCEC, STAD, and COAD, respectively, (Figure 4(b)), which had the capacity for inducing a frameshift mutation of the TLE3 gene, translating from A (Alanine) to T (Threonine) at the 477 sites of TLE3 protein. A477T site is shown in the 3D structure of the TLE3 protein (Figure 4(c)). Furthermore, the impact of TLE3 gene mutations on survival in pan-cancer was also studied. As indicated in Figure 4(d) from UCEC cases, we were unable to detect the association between alterations of TLE3 and OS, DSS, DFS, and PFS, compared with cases without altered TLE3.

### 3.5. DNA Methylation Analysis

**Figure 5:**
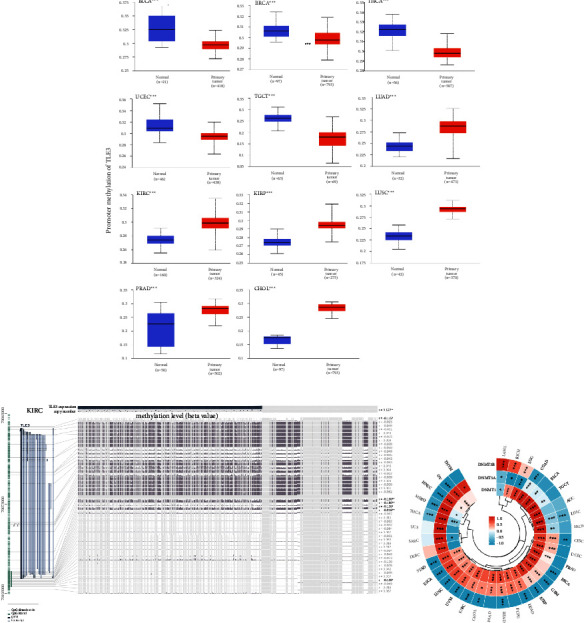
(a) Comparison of DNA methylation of TLE3 in normal and tumor tissues.  ^*∗*^*P* < 0.05;  ^*∗∗*^*P* < 0.01;  ^*∗∗∗*^*P* < 0.001, (b) association between TLE3 DNA methylation and gene expression for the KIRC, and (c) the relationship between TLE3 mRNA expression and MMR genes.

DNA methylation of TLE3 was investigated using the UALCAN database. The TLE3 expression of BLCA, BRCA, THCA, UCEC, and TGCT were significantly downregulated compared to normal tissues. The TLE3 methylation level in LUAD, KIRC, KIRP, LUSC, PRAD, and CHOL were greatly increased (Figure 5(a)). However, no differences were observed in other cancer tissues and matched normal tissues (Figure S2). We then explored the association between TLE3 DNA methylation sites and its mRNA expression based on MEXPRES and found an inverse relationship between TLE3 DNA methylation and mRNA expression of many probes in the KIRC nonpromoter region (Figure 5(b)). We next analyzed the correlations between TLE3 mRNA expression and DNA methyltransferases(DNMT1, DNMT2, DNMT3A, and DNMT3B, Figure 5(c)), and found that TLE3 expression was strongly correlated with DNA methylation in different cancers, particularly in LAML, KICH, LGG, COAD, LIHC, CESC, GBM, KIRP, LUAD, READ, KIRC, UVM, LUSC, ESCA, STAD, THCA, MESO, HNSC, and OV.

### 3.6. Protein Phosphorylation Analysis

**Figure 6:**
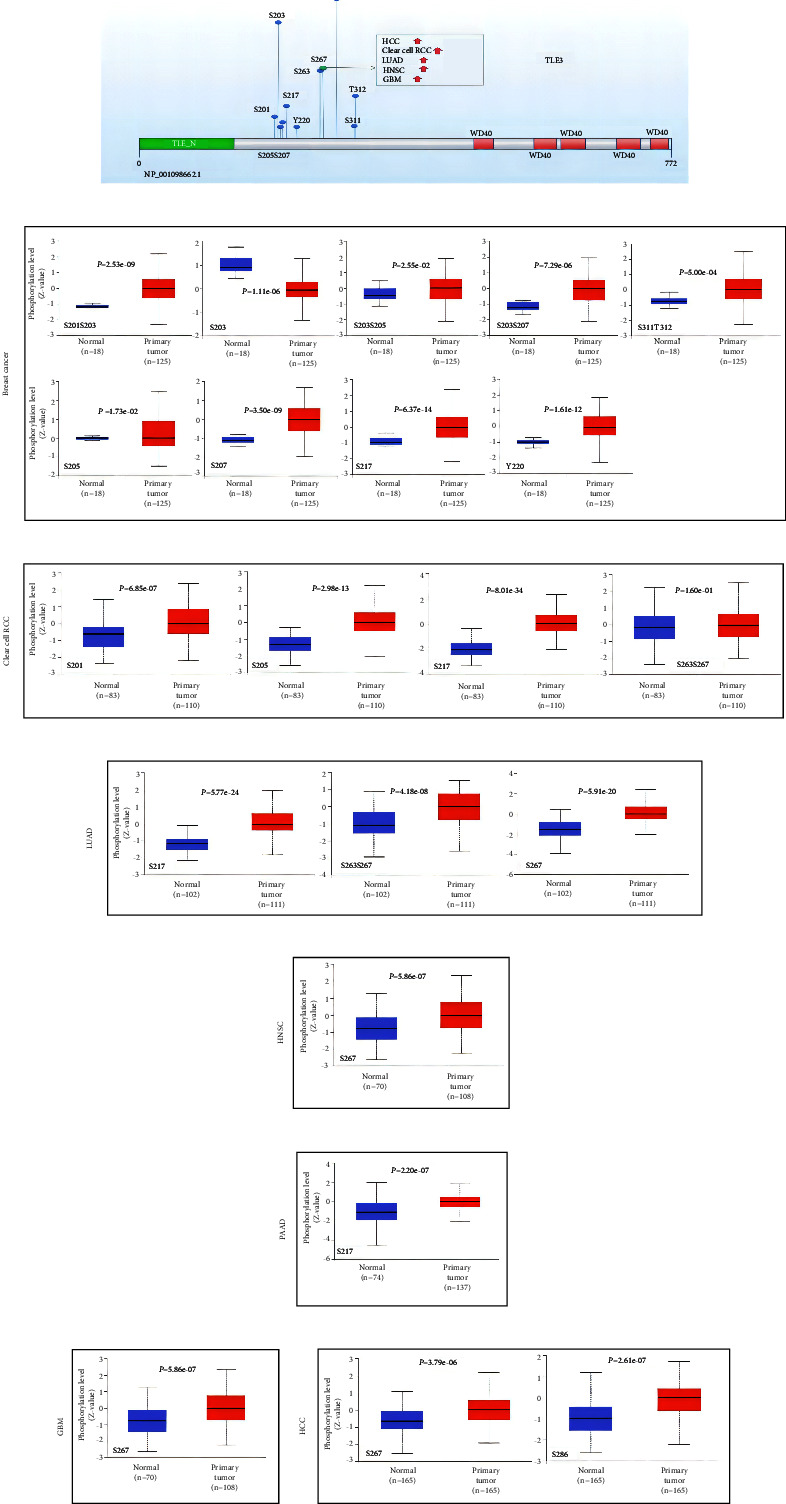
Protein phosphorylation analysis of Tle3 in different tumors. The expression of the Tle3 phosphorylation (NP_001098662.1, S267, S201S203, S203, S203S205, S203S207, S205, S207, S217, Y220, S311T312, S267, S286, S201, and S263S267) between primary tissue of selected tumors and normal tissue. (a) Schematic representation of the phosphoprotein sites of TLE3 detected based on the CPTAC dataset (b)–(h) box plot representing TLE3 phosphoprotein levels in intermediate and normal tissues of breast cancer, clear cell RCC, LUAD, HNSC, PAAD, GBM, and HCC.

Next, we compared the differences in TLE3 protein phosphorylation levels between the seven primary tumor tissues and the corresponding normal tissues from the CPTAC dataset and developed a schematic representation of the TLE3 phosphorylation sites in Figure 5(a). Phosphorylation levels of S267 were higher in most primary tumor tissues than in normal tissues, including HCC, LUAD, GBM, and HNSC. Moreover, the S217 phosphorylation level was increased in four cancers (Figures 6(a)–6(g)). The implication of these results was that high-level phosphorylation sites of Tle3, such as S267 and S217, may promote cancers and it should be further explored.

### 3.7. Immunotherapeutic Response of TLE3

**Figure 7:**
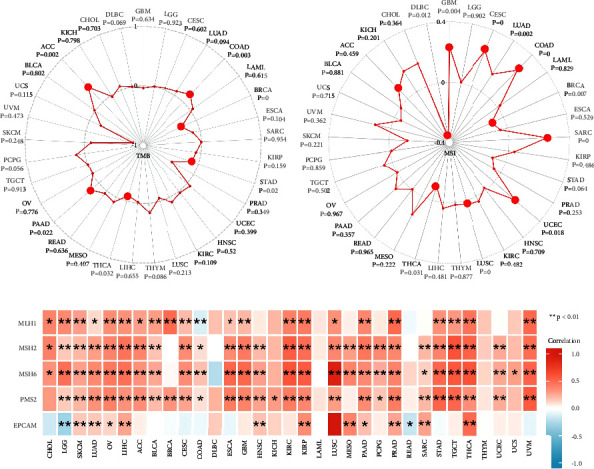
(a) The latent correlation between TLE3 mRNA expression and TMB, (b) the latent correlation between TLE3 mRNA expression and MSI, and (c) the latent correlation between TLE3 mRNA expression and MMR.

According to Figure 1, we observed high expression of TLE3 in bone marrow and neutrophils, indicating that it may play an antitumor role in immunity. Therefore, we explored the relationship between TLE3 mRNA expression and the biomarkers TMB, MSI, and MMR that reflect the immune response. TLE3 was significantly associated with TMB in COAD, BRCA, STAD, THCA, PAAD, and ACC (Figure 7(a)). The high TLE3 mRNA expression was correlated with higher TMB in COAD, PAAD, and ACC, indicating a better immunotherapy potential. There was a significant relation between TLE3 mRNA expression levels and MSI, CESC, LUAD, COAD, BRCA, SARC, UCEC, LUSC, THCA, ACC, and DLBC (Figure 7(b)). We also studied the effect of TLE3 mRNA expression on MMR genes, including MLH1, MSH2, MSH6, PMS2, and EPCAM. TLE3 was significantly related to all MMR genes in nine cancers, including LGG, SKCM, LUAD, OV, LIHC, KIRP, PAAD, PRAD, and THCA (Figure 7(c)). These results suggested that TLE3 could influence the immunotherapy response of different types of cancers.

### 3.8. Immunocorrelation Analysis of TLE3

**Figure 8:**
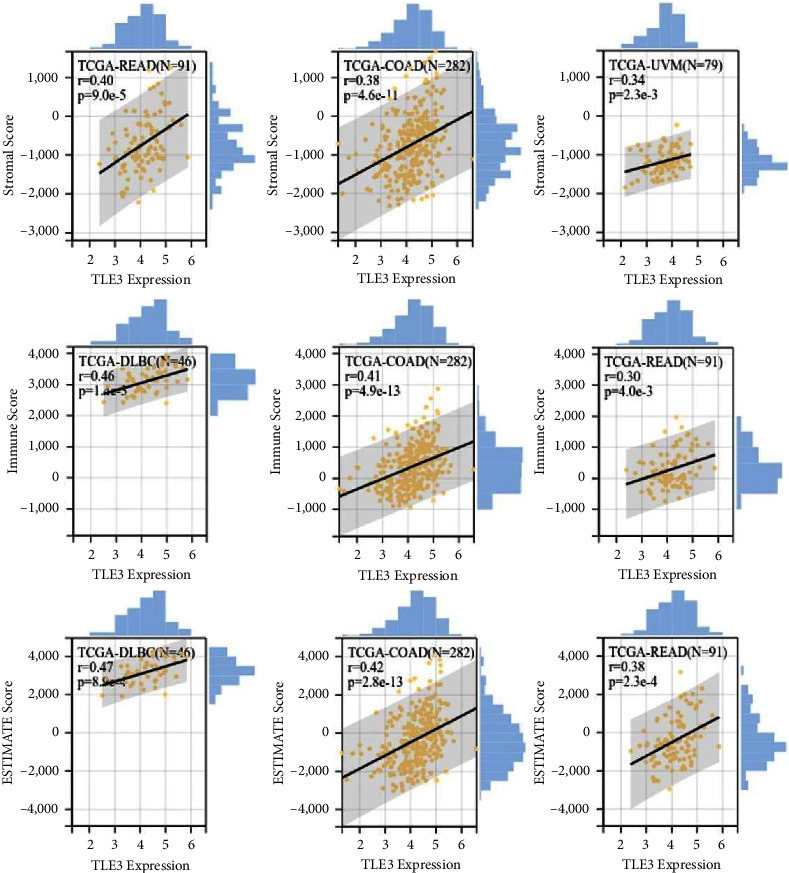
The top three tumors with the most significant correlation between the degree of immune infiltration and Tle3 mRNA expression were READ, COAD, and UVM (StromalScore); DLBC, COAD, and READ (ImmuneScore); and DLBC, COAD, and READ (ESTIMATEScore), respectively.

Tumor-infiltrating immune cells not only disrupt cytokine signaling pathways in the tumor microenvironment, but also play an important part in cancer initiation, progression, or metastasis [21, 22]. The top three tumors the most significantly positively associated with TLE3 expression were TGCT, LUSC, and BRCA (StromalScore), LUSC, UCEC and SKCM (ImmuneScore), LUSC, UCEC and SKCM (ESTIMATEScore), respectively (Figure 8).

**Figure 9:**
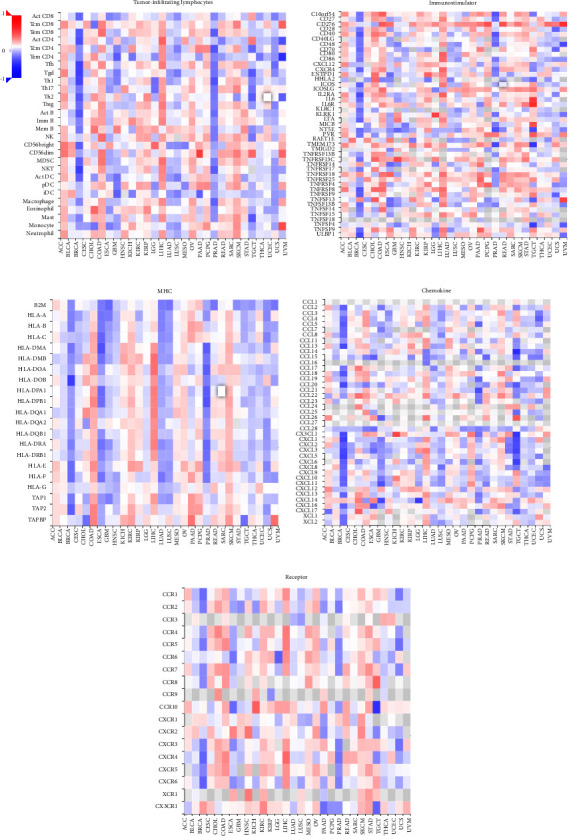
According to the tumor-immune system interactions and drug bank (TISIDB) database, the Spearman correlations between TLE3 mRNA expression and tumor-infiltrating lymphocytes (a) immunostimulatory, (b) MHC molecules, (c) chemokines, and (d) receptors.

To further study the TLE3 effect on immunological status in pan-cancers, we analyzed the association between its mRNA expression and the immunomodulators in 33 cancer types using the TISIDB database. As illustrated in Figure 9, our findings revealed that TLE3 was negatively correlated with most of the immunomodulators in BRCA but positively correlated with LIHC. In addition, the MCPcounter algorithm was used to evaluate the immune infiltration of TLE3 in different cancers to verify the above results. The current results consistently showed that TLE3 was positively correlated with the immune infiltration of LIHC (Figure S5).

**Figure 10:**
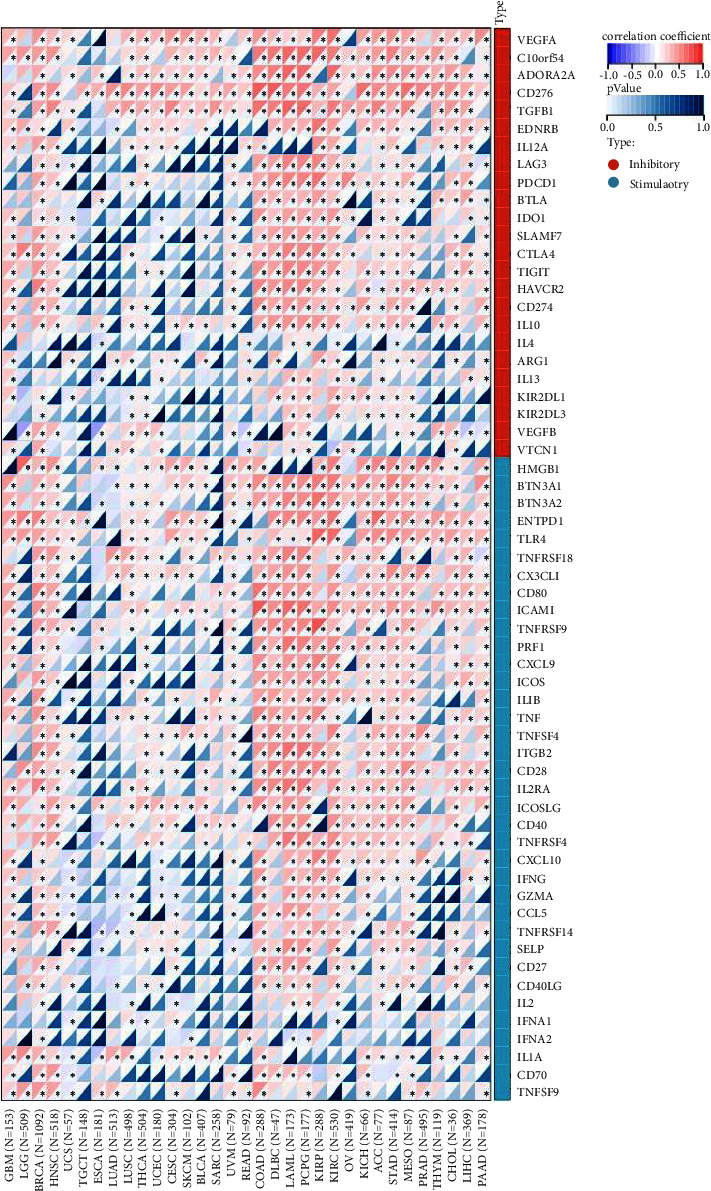
mRNA expression relationship between TLE3 and 60 immune checkpoints.

We also used the data from the TCGA database to evaluate the relationship between the TLE3 expression and 60 checkpoint genes (Figure 10). Analysis of TLE3 correlation with checkpoint gene expression in pan cancers showed that Tle3 was closely linked to CD276. Moreover, the TLE3 expression was positively related to a large number of immune checkpoint genes in many cancers, especially KIRC, BRCA, and LAML, suggesting that TLE3 participated in the tumor immune response regulation through modulating immune checkpoint activity.

### 3.9. Enrichment Analysis of TLE3

**Figure 11:**
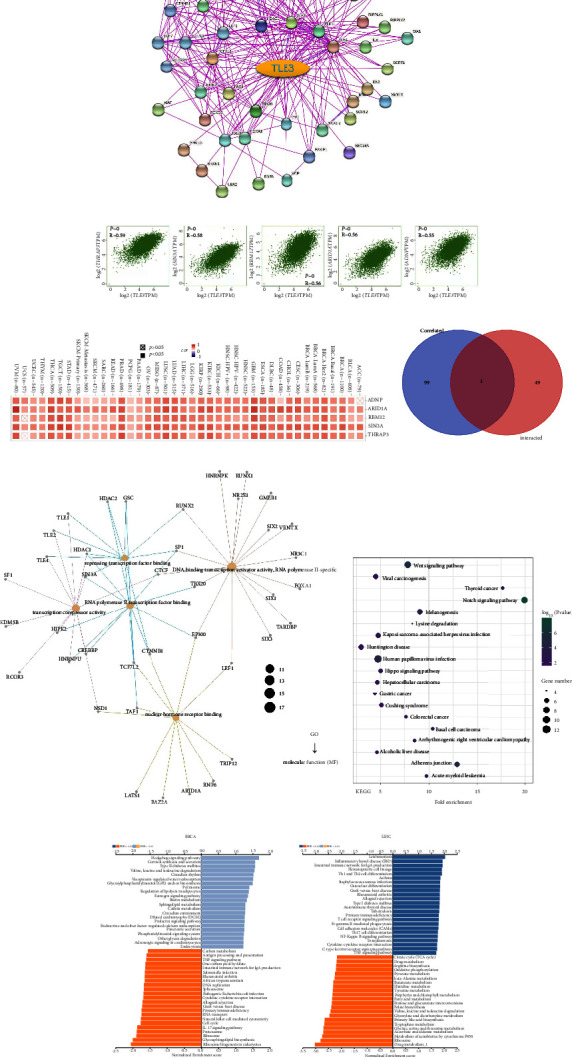
(a) Available experimentally determined tle3-binding proteins obtained using the String tool. Based on the STRING tool, we obtained the available experimentally determined Tle3-binding proteins, (b) the top 100 genes associated with Tle3 in the TCGA project were obtained by the GEPIA2 method, and the expression correlation between Tle3 and the selected targeted genes, including THRAP3, SIN3A, RBM12, ARID1A, and ADNP, were analyzed, (c) the heatmap shows the detailed cancer types, (d) an intersection analysis between the Tle3-binding and correlated genes, (e) KEGG analysis based on the Tle3-binding and interacted genes, (f) GO term enrichment analysis of Tle3-binding and interacted genes in molecular function, and (g) GSEA analysis of TLE3 showing in BRCA, LIHC using LinkedOmics data.

To elucidate the TLE3 gene molecular mechanism in tumorigenesis, a pathway enrichment analysis was performed after screening proteins bound to TLE3 targeting and those associated with expressed TLE3. Using the STRING tool, we detected 50 TLE3-binding proteins, all of which were experimentally validated. The network map of proteins interacting with TLE3 is shown in Figure 11(a). We also performed a data visualization analysis, as shown in Figure S3, TLE1, TLE4, TLE2, and TLE3 in the TLE family were the most closely related. The relationship between different genotypes and different subtypes, and genes was explored, and an interaction network was obtained (Figure S6). The top 100 genes associated with TLE3 expression were obtained from all tumor expression data from TCGA using the GEPIA2 tool. As shown in Figure 11(b), the expression level of *TLE3* was positively correlated with that of *THRAP3*, *SIN3A*, *RBM12*, *ARID1A,* and *ADNP* genes (all *P* = 0).  Figure 11(c) showed a positive relationship of TLE3 with the above five genes in 33 cancers. The intersection of the two groups was PAXIP1 (Figure 11(d)).

We performed KEGG and GO enrichment analyses combining the two datasets.  Figure 11(e) suggested KEGG data that the “Wnt signaling pathway,”” Notch signaling pathway,” and “Human papillomavirus infection” might be related to the effect of TLE3 on tumor pathogenesis. The GO data suggested that most of these genes were related to transcription factor binding (Figure 11(f)). The enrichment map obtained by Cytoscape Software also showed 23 enriched pathways, the top three with the highest enrichment were “Pathways in cancer,” “Human papillomavirus infection,” and “Wnt signaling pathway,” “Pathways in cancer” was associated with 17 of these pathways (Figure S4). In addition, we also studied BRCA and LIHC, respectively, two cancers with a strong correlation with TLE3. Furthermore, we also studied BRCA and LIHC, two cancers with a strong correlation with TLE3, separately, using the GSEA methods. We found that TLE3 was involved in immune-related pathways (Figure 11(g)).

## 4. Discussion

TLE3 properties in tumorigenesis have been found in addition to its dynamic functions in differentiation and cell metabolism [23–26]. To elucidate the molecular mechanism of TLE3 in cancers, we comprehensively analyzed the molecular characteristics of its mRNA and protein expression, gene mutation, DNA methylation, protein phosphorylation, and the immune microenvironment in 33 cancers based on available data from TCGA, GEO, and GTEx. According to our results, the TLE3 protein had a conserved structure in various species, indicating that effects of TLE3 may exist with similar mechanisms between these species, hence, it could be viable to use mice for studying TLE3-related cancer in humans. TLE3 is wildly distributed in many tissue types and immune cells, especially in bone marrow and neutrophils, which may be associated with immunity, and this further encouraged us to study its immunoprognostic value in tumors.

Numerous researches have reported that TLE3 expression is upregulated in multiple cancers, including cervical cancer [27] and malignant meningiomas [28]. TLE3 alternatively spliced isoforms have been detected to be upregulated in prostate tumors [7, 29], suggesting that the abnormal expression of TLE3 is strongly connected with cancer invasiveness. According to our results, TLE3 expression was high in most tumors with consistent results. The data for genetic alterations from TCGA and protein alterations from CPTAC were not entirely consistent, which may be resulted from the fact that different acquisition and calculation of the data in the database would be affected by gene mutations, promoter methylation, and protein phosphorylation to some extent. TLE3 was high-expressed in the pathological stage I gene of OV and OV patients with high expression of TLE3 had a poor prognosis, suggesting that the gene played a role as an oncogene in OV, and the OV patients can be treated in the early stage or separately according to their pathological stage.

GEPIA2, Cox regression, and Kaplan–Meier analysis indicated that the prognosis of ACC with high TLE3 expression was poor. Besides, high concentrations of GRO homologous to the human gene TLE3 was found in ACC cells [30]. Cox regression and Kaplan–Meier analysis suggested that TLE3 was a protective factor for breast cancer. A recent study has shown that miR-3677 can accelerate cell proliferation, migration, and invasion by targeting TLE3 in human breast cancer [31]. Another study found that TLE3, a transcription corepressor recruited by FOXA2 to the ZEB2 promoter, inhibits the expression of the EMT-related transcription factor ZEB2, thereby suppressing the EMT of breast cancer cells [32]. The results of different studies were consistent with our survival data. These findings strongly supported that TLE3 may be a potential prognostic indicator and molecular therapeutic target in ACC and BRCA.

We explored the immune characteristics of TLE3 in the tumor microenvironment (TME), including tumor-infiltrating lymphocytes, immunostimulatory factors, MHC molecules, chemokines, receptors, and immune checkpoints. Additionally, three immunotherapeutic biomarkers (TMB, MSI, and MMR) were found to be significantly associated with TLE3 in some cancers. Our results showed that the poor prognosis of ACC with high TLE3 expression was positively related to TMB and MSI. Immune checkpoint therapy (ICT) to enhance the antitumor immune response of T cells is a novel treatment for malignant tumor. CD276 is an immune checkpoint molecule in the epithelial-mesenchymal transformation (EMT) pathway, which plays a crucial part in the cell proliferation, invasion, and migration of malignant tumors, and is a promising therapeutic target for tumors [33]. Here in this study, we also found that TLE3 was positively associated with many immune checkpoint molecules, indicating that it had the potential to become a novel immunotherapy target. It is worth noting that TLE3 was high-expressed in BRCA and negatively related with TMB, MSI, as well as most of the immune regulatory factors in BRCA. Therefore, we hypothesized that TLE3 played an immunosuppressive role in BRCA and may be an additional diagnostic marker and therapeutic target. TLE3 was positively associated with most of the immunomodulators of LIHC, indicating that LIHC was a candidate cancer type suitable for TLE3 immunotherapy. In conclusion, TLE3 played an important and dual role (both inhibition and promotion) in pan-cancer immune infiltration.

Furthermore, the PPI map obtained by Cytoscape software suggested that TLE3 may play a role together with the TLE family in tumors. We performed a functional enrichment analysis of TLE3, and confirmed the potential effects of the “Wnt signaling pathway” and “Notch signaling pathway.” It has been found that Wnt signaling plays a role in multiple immune cells, including in DCS, NK cells, T cells, B cells, and also macrophages [34], and the Wnt/beta-catenin pathway can regulate the immune response against cancer [35]. Notch signaling also has critical functions in cancer, including the generation of blood vessels, the maintenance of cancer stem-cell-like cells, and tumor immunity [36]. Moreover, Enriched Map by Cytoscape Software also showed “Pathway in cancer,” pointing to the close potential relationship between TLE3 and cancers. KEGG and GO enrichment and GSEA analysis of BRCA and LIHC also confirmed the correlation between TLE3 and the immune microenvironment, suggesting the significance of TLE3 in the immunity of a variety of cancers.

In brief, our study showed that TLE3 was high-expressed in the majority of cancers, had different prognostic effects in various tumor cases, and was associated with the progression of OV. The TLE3 expression was also associated with TME. The above data suggested that TLE3 was a valuable novel biomarker for prognostic and immunotherapy response assessment in several cancers.

However, our study has certain limitations. First of all, some of our results were obtained from the bioinformatics website as we did not have a complete grasp of the *R* language. Secondly, this manuscript was based on bioinformatics research, the results were inconsistent sometimes due to differences in algorithms and databases. Therefore, the current findings should be verified experimentally, especially the role of TLE3 in BRCA and LIHC.

## Data Availability

The data used to support the findings of this study are included in the article.

## Supplementary Materials

Supplementary Materials Figure S1 mRNA expression states and protein level of TLE3 in human tumors. (a) TLE3 mRNA expression level comparison in 7 cancers (TCGA project) relative to the corresponding normal tissues (GTEx database), all *P* > 0.05. (b) The stage-dependent expression level of TLE3. The main pathological stages (stage I, stage II, and stage III) of 23 cancers were assessed and compared by TCGA data, all *P* > 0.05. Figure S2 comparison of DNA methylation of TLE3 in normal and tumor tissues (all *p* > 0.05). Figure S3 PPI map obtained by Cytoscape software. The nodes in the figure represent the experimentally verified proteins binding to TLE3, and the node color represents the degree of nodes interacting with the node. The darker color of the node, the more pathways that depend on the node, and the more important the node is. Edges represent interactions between nodes. Figure S4 enrichment map obtained by Cytoscape Software. A node represents the gene set, the edge represents the overlap of gene members, and the darker the node color, the higher the enrichment degree. Figure S5 the immune infiltration of TLE3 in different cancers based on the MCPCOUNTER algorithm. Figure S6 the relationship between different tle genotypes and different subtypes and genes.
