# Supplementary material for: Pan-Cancer Analysis of TLE3 Revealed Its Value in Tumor Microenvironment and Prognosis
Source: J Oncol. 2022 Nov 21;2022:4085770. doi: 10.1155/2022/4085770 (PMC9705091; doi:10.1155/2022/4085770)
Supplement: Supplementary Materials — Figure S1 mRNA expression states and protein level of TLE3 in human tumors. (a) TLE3 mRNA expression level comparison in 7 cancers (TCGA project) relative to the corresponding normal tissues (GTEx database), all P > 0.05. (b) The stage-dependent expression level of TLE3. The main pathological stages (stage I, stage II, and stage III) of 23 cancers were assessed and compared by TCGA data, all P > 0.05. Figure S2 comparison of DNA methylation of TLE3 in normal and tumor tissues (all p > 0.05). Figure S3 PPI map obtained by Cytoscape software. The nodes in the figure represent the experimentally verified proteins binding to TLE3, and the node color represents the degree of nodes interacting with the node. The darker color of the node, the more pathways that depend on the node, and the more important the node is. Edges represent interactions between nodes. Figure S4 enrichment map obtained by Cytoscape Software. A node represents the gene set, the edge represents the overlap of gene members, and the darker the node color, the higher the enrichment degree. Figure S5 the immune infiltration of TLE3 in different cancers based on the MCPCOUNTER algorithm. Figure S6 the relationship between different tle genotypes and different subtypes and genes. [file 4085770.f1.zip › Supplemental Description (1).docx]

**Supplemental Description**

Figure S1 mRNA Expression states and protein level of TLE3 in human tumors. **(a)** TLE3 mRNA expression level comparison in 7 cancers (TCGA project) relative to the corresponding normal tissues (GTEx database), all *P* > 0.05. **(e)** Stage-dependent expression level of TLE3*.* Main pathological stages (stage I, stage II and stage III,) of 23 cancers were assessed and compared by TCGA data,all *P* > 0.05.

Figure S2 Comparison of DNA methylation of TLE3 in normal and tumor tissues (all p>0.05).

Figure S3 PPI map obtained by Cytoscape software.The nodes in the figure represent the experimentally verified proteins binding to TLE3, and the node color represents the Degree of nodes interacting with the node. The darker color of the node, the more pathways that depend on the node, the more important the node is.Edges represent interactions between nodes.

Figure S4 Enrichment Map obtained by Cytoscape Software.

Node represents the gene set, edge represents the overlap of gene members, and the darker the node color, the higher the enrichment degree.

Figure S5 the immune infiltration of TLE3 in different cancers ) based on the MCPCOUNTER algorithm.

Figure S6 the relationship between different tle genotypes and different subtypes, genes.
